# Effects of Hazard Types on Hazard Perception and Decision-Making Among Adolescent Bicyclists: Results of a Hazard Prediction Task

**DOI:** 10.3390/bs16050748

**Published:** 2026-05-12

**Authors:** Jiatong Guo, Longyilin Xu, Long Sun

**Affiliations:** School of Psychology, Liaoning Normal University, Dalian 116021, China; 17824775557@163.com (J.G.); 15140812011@163.com (L.X.)

**Keywords:** middle school students, hazard perception, decision-making, hazard type, adolescent bicyclists

## Abstract

Hazard perception (HP) is a critical component of road safety; however, the HP abilities of adolescent bicyclists (aged 13–16 years) and their associations with decision-making (DM) across different hazard types remain underexplored. This study investigated grade-related differences in HP and DM among adolescent bicyclists using a video-based hazard prediction task. A total of 115 middle school students who ride bicycles on a regular basis were recruited. A 3 (grade: 7th, 8th, and 9th) × 2 (hazard type: environmental prediction hazards/EPs, behavioural prediction hazards/BPs) mixed experimental design was employed. Participants answered two HP questions (What is the hazard? What happens next?) and one DM question (What would you do?). Students in the ninth and eighth grades had higher scores on BP hazards in the “what happens next” and DM questions than did their peers in the seventh grade. Although students in the ninth grade made more safe decisions on the EP hazards than their peers in the seventh and eighth grades, their scores on the “what happens next” question were similar on EP hazards. Importantly, students in the ninth grade scored significantly higher on DM than on HP for EP hazards, while students in the eighth grade scored significantly higher on DM than on HP for BP hazards. These findings demonstrate that both HP and DM accuracy improve with grade level and that the relations between HP and DM varies by hazard type. The results underscore the necessity for targeted, hazard-specific training programmes to improve cycling safety among adolescents.

## 1. Introduction

The ability to anticipate potential hazards, defined as hazard perception (HP), is closely related to the occurrence of traffic crashes (Horswill et al., 2015). Among the various tools developed to assess HP across road users, the hazard prediction task stands out as one of the most widely employed. Compared with traditional response-time-based tests, the hazard prediction test provides a more accurate assessment of hazard perception by reducing response bias (Crundall, 2016). In this test, participants view video clips recorded from a bicyclist’s perspective, each ending abruptly with a black screen, and are subsequently asked to identify the potential hazard, its location, and its likely outcome (e.g., Castro et al., 2014; Gugliotta et al., 2017). The main body of literature has demonstrated that the hazard prediction test effectively distinguishes drivers with varying levels of experience (e.g., Castro et al., 2021; Gugliotta et al., 2017) and differentiates high- from low-risk drivers (e.g., Castro et al., 2014; Wu et al., 2021). More recently, its applicability and validity have been extended to e-bike riders and bicyclists (e.g., Kovácsová et al., 2020; Sun et al., 2023).

In this study, a bicycle is defined as a non-electric, human-powered, two-wheeled vehicle with low speed (Fishman & Cherry, 2016). Safe bicycling depends on the coordinated use of motor and cognitive skills, both of which develop with experience and maturation. The ability to operate a bicycle and pay attention to potential hazards in the traffic environment affects the crash involvement of bicyclists (Sun et al., 2023). Timeliness and appropriateness of DM, including judgments about whether to brake, swerve, or maintain one’s current course, are equally critical to avoiding collisions. Given that both HP and DM are deeply intertwined and embedded in real-world cycling contexts, it is essential to investigate how they co-occur and interact across different hazard scenarios. However, research in this area still lacks empirical evidence, particularly among adolescent bicyclists, a population whose perceptual and decision-making capacities are still developing. To address this significant gap, the present study employs a video-based hazard prediction task to simultaneously assess HP and DM among adolescent bicyclists, with the aim of clarifying how hazard type shapes these two processes and informing evidence-based interventions targeting cycling safety.

### 1.1. Adolescent Bicyclists: A Vulnerable and Understudied Population

Although Chinese law permits bicycling from age 12 (coinciding with middle school entry), adolescent bicyclists aged 12–18 years experience a disproportionately high rate of traffic crashes (National Health Commission of China, 2020). In 2023 alone, China reported an estimated 400 million bicycles in circulation and 3119 bicycle-related traffic crashes, resulting in 554 fatalities and 3090 injuries (National Bureau of Statistics of China, 2023). These figures underscore the urgent need to comprehensively understand the perceptual and behavioural factors that contribute to crash risk in this population. To date, however, research has predominantly focused on either child bicyclists (aged 9–11) or adults, leaving adolescents as a comparatively underexplored group. While child bicyclists are less capable of timely hazard detection and safe decision-making relative to adults, owing largely to their limited riding experience (Zeuwts et al., 2017), adolescent bicyclists occupy an intermediate position; although they possess greater experience than children, their situational awareness typically remains inferior to that of adults (Zeuwts et al., 2017). This gap is partly attributable to ongoing neurocognitive development, as brain regions responsible for executive functions, impulse control, and future-oriented thinking continue to mature throughout adolescence (Casey et al., 2008). Consequently, older adolescents gradually refine their capacity to anticipate hazards and make adaptive decisions, particularly as they accumulate cycling experience (McCartt et al., 2009). Given that these perceptual and decision-making capacities are still developing during this period, it is essential to investigate how adolescent bicyclists respond to different types of traffic hazards and how such responses evolve across grade levels.

Evidence from driver studies suggests that hazard types impose different cognitive demands (Crundall et al., 2012). Despite this, no study to date has systematically compared how adolescent bicyclists perform across these two hazard categories in terms of both HP and DM, representing a significant gap in the literature given that adolescents’ still-maturing perceptual and executive capacities may render them differentially vulnerable to the cognitive demands each hazard type imposes.

### 1.2. Hazard Perception in Adolescent Bicyclists: EP Versus BP Hazards

Several studies have examined the HP abilities of adult and child bicyclists using video-based hazard prediction tests. Vansteenkiste et al. (2016) found that adults reacted to hazards more quickly than eight-year-old children, while Sun et al. (2023) reported that adults aged 18–30 years outperformed adolescents aged 13–16 years in hazard prediction accuracy. Taken together, these findings consistently suggest that HP abilities improve with age and experience. Nevertheless, despite this growing body of evidence, relatively little is known about the HP abilities of adolescent bicyclists who regularly commute to and from school, a subgroup whose routine exposure to real-world traffic environments may meaningfully shape their perceptual development in ways that have yet to be systematically examined.

The present study classifies hazards into two distinct types, following the framework originally developed in driver research (Crundall et al., 2012) and subsequently adapted for bicyclists (Zeuwts et al., 2017). BP hazards involve visible road users who, given the circumstances, may begin to act dangerously (e.g., a bicyclist riding straight when a nearby car door suddenly opens). EP, by contrast, involve potential collisions with road users who are entirely concealed by stationary objects, such as a pedestrian stepping unexpectedly into the road from behind a stopped bus (Crundall et al., 2012). This distinction is not merely descriptive but reflects fundamentally different cognitive demands; whereas BP hazards call for immediate detection and rapid motor response, EP hazards require higher-order predictive reasoning, a working knowledge of traffic rules, and a degree of situational awareness that accrues gradually through experience (e.g., Crundall et al., 2012; Zeuwts et al., 2017). Consistent with this, evidence from both driving and cycling research indicates that EP hazards are considerably more perceptually demanding than BP hazards, precisely because they cannot be directly observed and must instead be inferred from subtle contextual cues in the environment (e.g., Crundall et al., 2012; Zeuwts et al., 2017). Whether this pattern extends to adolescent bicyclists, as well as whether the relative difficulty of EP hazards attenuates across grade levels as cognitive capacities mature and cycling experience accumulates, remains an open empirical question that the present study directly seeks to address.

### 1.3. Relationships Between Hazard Perception and Decision-Making

To the best of our knowledge, no study has yet explored the relationships between the HP abilities and DM among adolescent bicyclists aged 13–16 years, representing a critical gap in the existing literature. To date, research on this association has focused predominantly on drivers (Castro et al., 2021, 2024; Gugliotta et al., 2017), with only limited attention to bicyclists and virtually none to adolescents. Utilizing a video-based hazard prediction task, Gugliotta et al. (2017) reported that novice drivers were less adept at recognizing hazards than their experienced counterparts and that DM scores consistently exceeded HP scores. Castro et al. (2021) further showed that riskier decisions were associated with poorer HP, a relationship that was pronounced among novice drivers and non-drivers. More recently, Castro et al. (2024) replicated these findings among drivers with stroke-related cognitive impairments, further supporting the notion that HP and DM are related but dissociable constructs. Given that hazard perception represents merely the perceptual precondition for safe cycling, effective road behaviour ultimately depends on whether the bicyclist can also formulate and execute a timely and appropriate response; understanding this two-stage process among adolescent bicyclists is therefore essential for developing effective road safety education programmes in Chinese middle schools.

### 1.4. The Present Study

The hazard prediction test is grounded in situational awareness theory, which comprises three levels: perception, comprehension, and prediction (Endsley, 1995). The test offers a suitable framework for examining how different hazard types interact with developmental stages. Well-developed situational awareness is crucial for different road users to foresee hazards and choose appropriate responses (Gugliotta et al., 2017; Zeuwts et al., 2017). Given that adolescents are still in the process of developing key cognitive functions, including sustained attention, anticipatory reasoning, and impulse control, this framework is especially well suited to capturing age-related differences in HP and DM across hazard types. However, despite its theoretical relevance, SA theory has rarely been applied systematically to adolescent bicyclists, and the extent to which each level of SA differentially predicts DM outcomes across EP and BP hazards remains empirically unexamined.

The present study therefore aimed to examine hazard perception and decision-making among adolescent bicyclists aged 13–16 years using a video-based hazard prediction task. First, how do hazard perception and decision-making accuracy differ between EP hazards and BP hazards among adolescent bicyclists in grades 7, 8, and 9? Second, does the relationship between hazard perception and decision-making accuracy vary by hazard type and grade level? By addressing these questions, this study advances the understanding of the cognitive mechanisms underlying cycling safety among adolescents, while simultaneously providing empirical evidence to support the design of tailored, hazard-specific road safety education programmes for middle school students in China.

## 2. Methods

### 2.1. Participants

A total of 130 middle school students aged 13–16 years who primarily relied on bicycles as their daily mode of transportation were recruited to participate in this study. The participants were recruited from one middle school located in Tianjin. Fifteen students declined participation after being informed of the purpose of this study, resulting in a final sample of 115 participants (61 males, 54 females). In total, 40 students were in the 7th grade, 38 were in the 8th grade, and 37 were in the 9th grade. With respect to bicycling frequency, 35 students (30.4%) rode a bicycle 5–6 days per week, 67 (58.3%) rode a bicycle 3–4 days per week, and 13 (11.3%) rode a bicycle 1–2 days per week. The demographic information of the participants is presented in Table 1.

### 2.2. Materials

#### 2.2.1. Hazard Prediction Test

**Figure 1:**
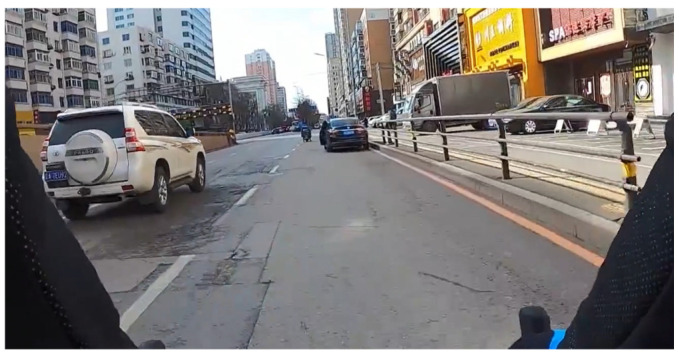
Screenshot of a hazardous video of BP hazard (a black car that is parked in our lane is suddenly opening the left side door).

**Figure 2:**
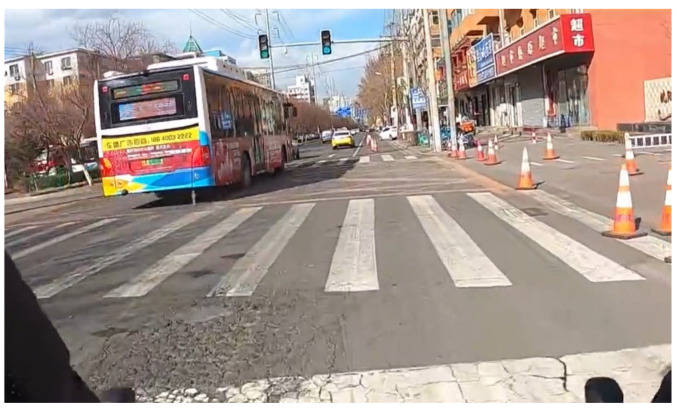
Screenshot of a hazardous video of EP hazard (a bus obscures a pedestrian at the zebra crossing).

Sixteen video clips drawn from a hazard prediction test developed by Sun et al. (2023) were used in this study. The test showed acceptable reliability in adult and child bicyclists. The lengths of the clips varied from 5 s to 9 s, with an average of 6.56 s (SD = 1.36 s). The scenarios depicted in the videos included potential collisions involving cars, buses, and pedestrians that could occur if the bicyclists did not execute evasive actions (see Figure 1 and Figure 2). The video scenarios used in the hazard prediction test cover both BP hazards and EP hazards (Crundall et al., 2012). Detailed descriptions of the hazards in the video clips are presented in the Supplementary Materials.

Three dependent variables were assessed in this study, consistent with the measurement approach adopted by Gugliotta et al. (2017). These comprised hazard identification (“What is the hazard?”; hereafter What), hazard anticipation (“What happens next?”; hereafter Whn), and decision-making (“What would you do in this situation?”; hereafter DM). Prior to completing the task, participants were informed that a hazard is defined as any obstacle or object requiring a behavioural response, whether braking or executing a manoeuvre to avoid a collision, consistent with the operational definition employed in previous research (e.g., Castro et al., 2019; Crundall, 2016). Following each video clip, participants were asked to respond to all three questions in sequence. Each response was scored dichotomously, with one point awarded for a correct answer and zero points for an incorrect answer.

Examples of the scoring criteria for Figure 1 are as follows. For the “what” question, the participants are required to report whether they have detected a hazard. If the answer is “no”, they receive 0 points and proceed to the next clip. If the answer is “yes”, they need to identify the hazard source (e.g., the white van in the left driving lane or just “van”). A correct answer earns 1 point, whereas other answers earn 0 points. For the “what happens next” question, the participants are provided with four choices, and they need to select only one choice that they think is correct by typing the number of their choice on the keyboard. In this study, scores on this question served as the HP score. A correct description earns 1 point, whereas an incorrect answer receives 0 points (e.g., 1 the white van in the left driving lane indicates that it will enter the driving lane; 2 the white van in the left driving lane will stop to yield to the bicycle; 3 the pedestrian on the right will cross the road; and 4 the bicycle in front will turn right). In line with the approach established in previous studies (Castro et al., 2014, 2019; Muela et al., 2021; Ventsislavova & Crundall, 2018), the response options for the multiple-choice questions were developed from the most frequent responses given by a sample of participants aged 13–16 years who took part in our previous hazard prediction test study when the same questions were presented in an open format (Sun et al., 2023). For the “DM” question, four options are provided to participants (at this point, you will 1 slow down or brake, 2 maintain the same speed and direction, 3 accelerate, or 4 change riding lane). If the given answer is a safe action (1 or 4), they receive 1 point; if they choose a risky action (2 or 3), they earn 0 points. The scoring criteria for each video were established through consensus between one expert in traffic psychology and one experienced adult bicyclist.

Adopting the method used by Gugliotta et al. (2017), this study clarified the “what happens next” question to distinguish it from the DM question. The participants were instructed to disregard any specific actions that they might take if they were the bicyclist and, instead, to concentrate on anticipating the behaviour of other road users within the traffic scenario or any consequential events that might subsequently unfold.

#### 2.2.2. Self-Reported Demographic Questionnaire

The participants’ demographic data, responses to the two HP questions and the DM question were collected. The demographic data collected included gender, age, and bicycling data (including bicycling frequency and distance). To control for the potential effects of confidence in bicycling, participants were also asked to rate their bicycling ability, awareness of others, and confidence in bicycling on a 7-point Likert scale.

### 2.3. Experimental Design and Hypotheses

A 3 (grade: 7th grade, 8th grade and 9th grade) × 2 (hazard type: BP hazard and EP hazard) mixed experimental design was adopted. Grade was treated as a between-groups factor, and hazard type was treated as a within-groups factor. The dependent variables were the scores on the two HP questions (what, whn) and the DM question. Based on the literature review presented above, two hypotheses were proposed:

**Hypothesis** **1.** *EP hazards are more difficult to detect than BP hazards among drivers with different levels of driving experience (e.g., Crundall et al., 2012; Sun & Hua, 2019). Accordingly, we predict that adolescent bicyclists will obtain higher hazard perception and DM scores on BP hazards rather than on EP hazards as their grade level increases.*

**Hypothesis** **2.** *Given that making correct decisions does not necessarily need to be based on complete situational awareness (Gugliotta et al., 2017), we predict that compared with EP hazards, adolescent bicyclists’ decisions about subsequent behaviours on BP hazards will be more accurate than hazard perception scores as their grade level increases.*

### 2.4. Procedure

Participants first completed the demographic questionnaire and the three self-report measures. Prior to the main experiment, they undertook a practice session comprising two video clips, during which they familiarized themselves with the task format by responding to all three questions. During the experiment, the participants viewed 16 randomly presented video clips on a 13.2-inch Huawei iPad with a resolution of 1920 × 1080 pixels. They were instructed to answer the three questions when each clip cut to a black screen before the hazard developed (Sun et al., 2023). The entire procedure lasted approximately 35 min, and each participant received monetary compensation of 15 RMB upon completion.

### 2.5. Data Analyses

Data analyses were conducted using SPSS 26.0. To test Hypothesis 1, which predicted that adolescent bicyclists would obtain higher HP and DM scores for BP hazards than for EP hazards as grade increases, the scores for the three questions were analysed using separate 3 (grade: 7th, 8th, and 9th) × 2 (hazard type: BP hazard and EP hazard) mixed ANOVAs. Hazard type was the only repeated-measures factor manipulated, and grade was the between-group factor. When interaction effects were identified, post hoc multiple comparison analyses (with Bonferroni correction) were conducted to determine which specific groups differed significantly from one another.

To test Hypothesis 2, which predicted that decisions regarding subsequent behaviours for BP hazards would be more accurate than HP scores as grade increases compared with EP hazards, a 2 (hazard type: BP hazard and EP hazard) × 2 (question type: HP and DM) × 3 (grade: 7th, 8th, and 9th) mixed ANOVA was conducted, in line with the method used in previous studies (e.g., Gugliotta et al., 2017). Question type and hazard type were treated as repeated measures and grade as the between-group factor. The hazard perception score was computed by averaging the scores of two hazard perception questions, while the DM score was based on the accuracy of the participant’s chosen action. However, at this time, the analysis only focused on the relationships between the HP ability and decision-making of students in each grade under different hazard types. The HP score was obtained by averaging the scores of two HP questions. Finally, the relationships between self-reported bicycling data and the scores on the three questions were analysed using Pearson correlation analysis to explore the extent to which cycling experience was associated with task performance. All assumptions underlying the mixed ANOVA were verified and satisfied for each dependent variable prior to analysis.

## 3. Results

### 3.1. What Is the Hazard?

**Figure 3:**
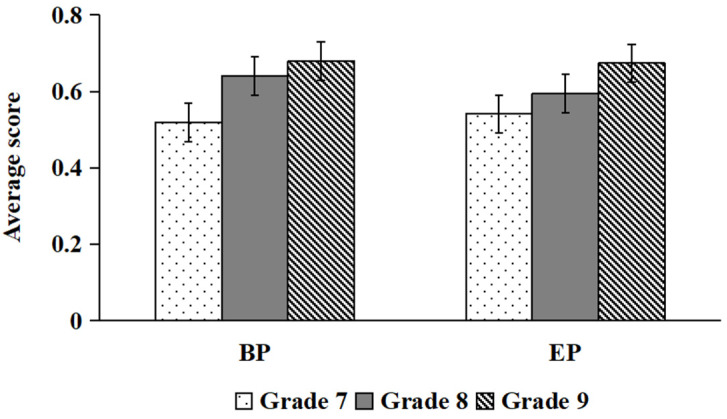
Comparisons of BP and EP scores for “what”.

There was a significant main effect of grade, *F*(2, 112) = 11.84, *p* < 0.01, *η*^2^ = 0.174, with ninth and eighth graders outperforming seventh graders. Neither the main effect of hazard type, *F*(1, 112) = 0.07, *p* = 0.79, nor the interaction effect between grade and hazard type, *F*(2, 112) = 0.90, *p* = 0.408, was significant (see Figure 3).

### 3.2. What Happens Next (Whn)?

**Figure 4:**
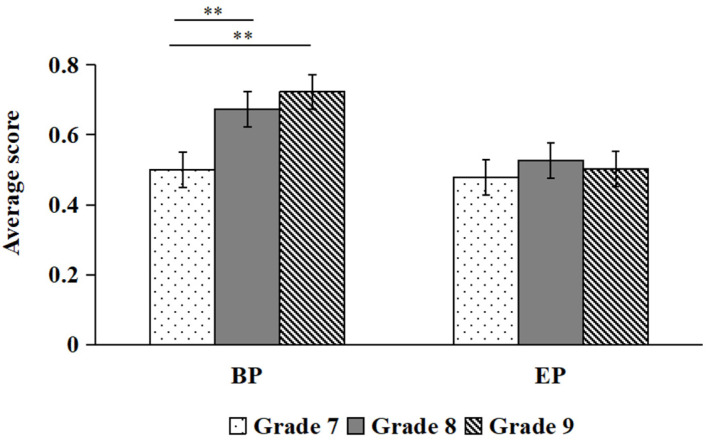
Comparisons of BP and EP scores for “whn” (** *p* < 0.01).

There was a significant main effect of grade, *F*(2, 112) = 10.34, *p* < 0.01, *η*^2^ = 0.156, with ninth and eighth graders scoring higher than seventh graders. The main effect of hazard type is significant, *F*(1, 112) = 44.45, *p* < 0.01, *η*^2^ = 0.284, with the scores for BP hazards higher than those for EP hazards. The interaction effect between grade and hazard type was likewise significant, *F*(2, 112) = 8.91, *p* < 0.01, *η*^2^ = 0.137. Simple effect analysis revealed that students in the ninth and eighth grades could better predict BP hazards than could students in the seventh grade (*ps* < 0.01), whereas no significant grade differences emerged for EP hazard prediction (*ps* > 0.05; see Figure 4).

### 3.3. Decision-Making

**Figure 5:**
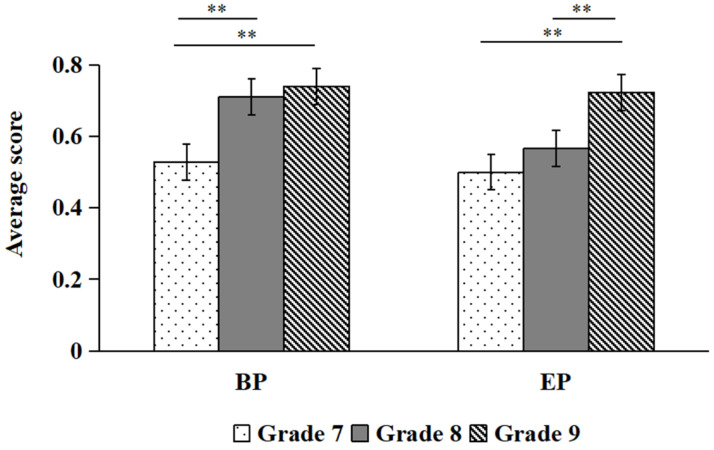
Comparisons of BP and EP scores for DM (** *p* < 0.01).

There was a significant main effect of grade, *F*(2, 112) = 26.26, *p* < 0.01, *η*^2^ = 0.319, with ninth graders scoring higher than seventh and eighth graders. The main effect of hazard type is significant, *F*(1, 112) = 7.88, *p* = 0.006, *η*^2^ = 0.066, with the scores for BP hazards being higher than those for EP hazards. The interaction effect between grade and hazard type was significant, *F*(2, 112) = 3.27, *p* = 0.042, *η*^2^ = 0.055. Simple effect analysis revealed that the scores of students in the ninth and eighth grades were higher on BP hazards than those of students in the seventh grade, *ps* < 0.01, while ninth graders scored higher than both seventh and eighth graders on EP hazards (*ps* < 0.01; see Figure 5).

### 3.4. Hazard Perception and Decision-Making

A 2 × 2 × 3 mixed ANOVA was conducted with question type, hazard type, and grade as independent variables and HP and DM scores as dependent variables. The main effect of grade, *F*(2, 112) = 25.61, *p* < 0.01, *η*^2^ = 0.314; hazard type, *F*(1, 112) = 16.85, *p* < 0.01, *η*^2^ = 0.131; and question type, *F*(1, 112) = 14.36, *p* < 0.01, *η*^2^ = 0.114, were found. The three-way interaction of question type, hazard type and grade was significant, *F*(2, 112) = 3.60, *p* = 0.030, *η*^2^ = 0.06.

**Figure 6:**
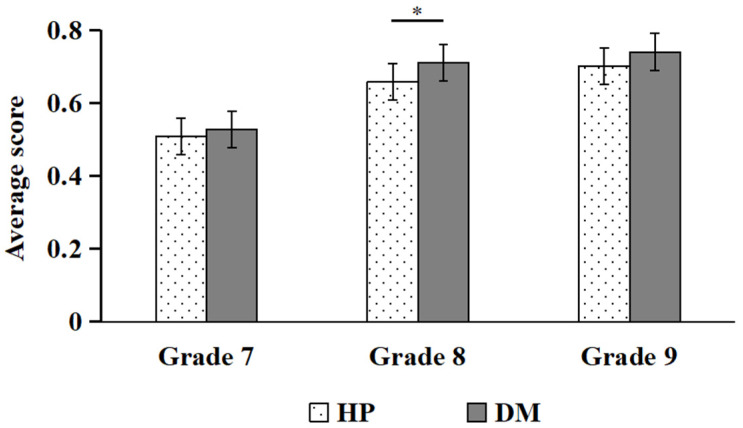
Comparisons of HP and DM scores under BP (* *p* < 0.05).

**Figure 7:**
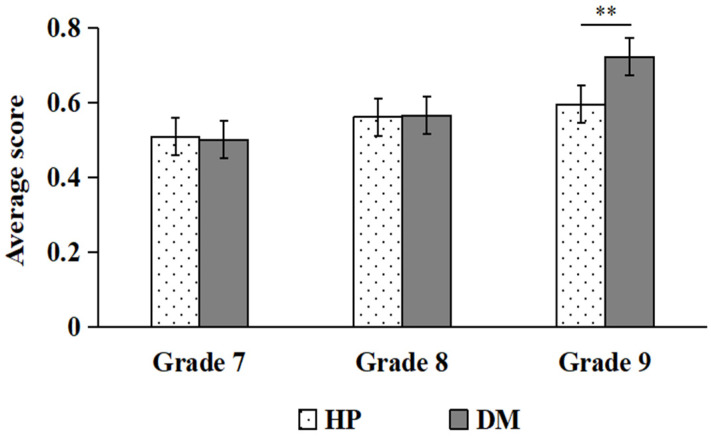
Comparisons of HP and DM scores under EP (** *p* < 0.01).

Simple effect analysis indicated that, for BP hazards, students in the eighth grade scored significantly higher on DM than on HP (*p* < 0.05; see Figure 6). For EP hazards, ninth graders scored significantly higher on DM than on HP (*p* < 0.01; see Figure 7).

### 3.5. Correlation Analyses

The correlations between bicycling-related data, participants’ subjective estimates of their bicycling ability, awareness of others and confidence in bicycling, and the scores of HP and DM under different hazard types are presented in Table 2.

Table 2 shows that bicycling frequency and bicycling distance were positively correlated with the scores on HP for both hazard types. Only the scores on DM for EP hazards were positively correlated with bicycling frequency. The score on HP for BP hazards was positively correlated with bicycling ability and self-confidence in bicycling, while the score on DM for BP hazards was positively correlated with awareness of others.

## 4. Discussion

To the best of our knowledge, this study is the first to explore the characteristics of HP and decision-making among adolescent bicyclists aged 13–16 years. Our findings show that hazard perception ability and decision-making performance of adolescent bicyclists increased with grades. Furthermore, hazard types affected the relationships between hazard perception ability and decision-making accuracy. Taken together, these findings advance theoretical understanding of the cognitive mechanisms underlying cycling safety in adolescence and carry direct implications for the design of targeted road safety education programmes in Chinese middle schools.

### 4.1. Effects of Grade and Hazard Type on Hazard Perception and Decision-Making

One notable contribution of this study is the confirmation of grade-related differences in HP ability and DM accuracy among adolescent bicyclists within the context of a video-based hazard prediction task. Specifically, the interaction between hazard type and grade level influenced hazard perception and decision-making among adolescent bicyclists. This study found that scores on outcome anticipation and DM were significantly higher among eighth- and ninth-grade students than among their seventh-grade counterparts, consistent with Hypothesis 1. These findings are further corroborated by the significant positive associations observed among cycling distance, cycling frequency, and task scores on HP and DM for BP hazards, lending empirical support to the argument that individual experience significantly shapes situational awareness (Underwood et al., 2013). Indeed, as adolescent bicyclists accumulate cycling experience, they are thought to develop richer mental templates encompassing a wider range of traffic scenarios and hazard types, progressively enhancing their capacity to respond adaptively to hazards and make increasingly accurate decisions (Kovácsová et al., 2020; Vansteenkiste et al., 2016; Zeuwts et al., 2017).

Although students in the ninth grade outperformed students in the seventh and eighth grades in the ability to make correct decisions on EP hazards, their scores on anticipation for EP hazards did not differ significantly across grade levels, suggesting that the ability to make safe decisions and the ability to accurately predict hazard outcomes may develop at different rates for this hazard type. A plausible explanation for this dissociation lies in the perceptual nature of EP hazards themselves. In this study, EP hazard clips depicted situations in which potential risks were fully concealed by stationary obstacles and had not yet materialized, which may have led adolescent bicyclists to underestimate the urgency of the situation and to fail to spontaneously anticipate the presence of a concealed road user. If adolescent bicyclists do not recognize that another road user may be obscured by an object in the environment, their capacity to anticipate a potential collision is fundamentally compromised, regardless of their general decision-making competence. These findings underscore the need for a nuanced, hazard-specific approach to traffic safety education in Chinese middle schools, with curricula tailored to address the distinct perceptual and cognitive demands imposed by EP and BP hazards across different grade levels.

Notably, regarding the question “What is the hazard?”, students in the eighth and ninth grades outperformed their seventh-grade counterparts, replicating the grade-related pattern observed for outcome anticipation. However, no significant difference between hazard types was found, suggesting that both EP and BP hazards were equally recognized by participants. This may indicate that adolescent cyclists have similar baseline recognition for both types of hazards, regardless of their complexity. This finding suggests that more advanced training in hazard perception focusing on the prediction and interpretation of hazard development could be more effective.

### 4.2. Hazard Perception Ability and Decision-Making Performance

A further contribution of this study concerns the hazard-type-specific relationships between HP and DM on EP hazards for students in the ninth grade, whereas such a pattern was not observed in seventh- or eighth-grade students. These results partially contradict Hypothesis 2 and may indicate that decision-making and hazard prediction are consciously related for students in the seventh and eighth grades. This hinted us that younger students should pay more attention to where exactly an EP hazard might appear and/or increase their awareness of the risk posed by the EP hazard to make the right decisions. These findings underscore the need for targeted HP training programmes that specifically address EP hazards for younger adolescent bicyclists, with particular emphasis on developing anticipatory reasoning skills and risk awareness among students in grades 7 and 8.

Furthermore, our findings support that only for students in the eighth grade, their decision-making on BP hazards is not fully contingent on their hazard perception ability. Notably, although no significant difference was found for students in the ninth grade, their scores on HP and DM for BP hazards were similar to those for students in the eighth grade. These findings imply that educational programmes targeting BP hazards may be of particular value for students in grade 7, who have yet to develop the perceptual and decision-making capacities observed in older peers. These results are supported by those of Gugliotta et al. (2017), who reported that individuals’ decision-making was somewhat not fully dependent on their situational awareness. Specifically, Gugliotta et al. (2017) explained that an individual’s decision-making is influenced by implicit processing (i.e., engaging in unconscious behaviours), and the decision-making process for behaviour can be summarized into only two types: (1) maintaining the original speed and direction or (2) changing speed and direction. In this regard, while accurate hazard prediction constitutes an essential foundation for safe cycling, the ability to execute a correct behavioural response may ultimately be more critical for collision avoidance. Nonetheless, the relationship between hazard perception and decision-making should be interpreted cautiously, and further research is needed to confirm and extend these findings.

### 4.3. Limitations and Future Research

Several limitations of the present study warrant consideration. First, hazard identification and outcome anticipation were assessed via self-report, which cannot entirely rule out the possibility of random guessing. Hence, incorporating physiological measures, such as eye-tracking or EEG, alongside self-report could provide a more comprehensive understanding of hazard perception processes in future studies. Second, the participants were recruited from a single middle school in Tianjin, which limits the generalizability of the findings to broader adolescent populations. Future research should include a more diverse sample from multiple schools across different regions to enhance the representativeness of the results. Third, by presenting video clips rather than real-world cycling scenarios, the present study necessarily decoupled perceptual skills from motor skills such as steering and pedalling. Future studies should explore the consistency between participants’ self-reported responses and their behavioural reactions to different hazards. Fourth, the absence of an adult comparison group limits the extent to which the present findings can be interpreted within a broader developmental framework. Future research should aim to include adult bicyclists as a comparison group to evaluate whether the hazard perception abilities and decision-making accuracy of adolescents differ from those of adults. Finally, the present study focused exclusively on hazard perception and decision-making in bicycling scenarios. The findings are specific to cycling-related hazards and cannot be directly generalized to other types of traffic hazards (e.g., pedestrian hazards, vehicle-only hazards) that adolescents may encounter in daily life. Future research should therefore examine whether the observed relationships between HP and DM accuracy extend to non-cycling hazardous situations in adolescents’ daily traffic environments.

Post-experiment interviews revealed that most student participants had not received any systematic traffic safety education, and no existing curriculum specifically addressed HP training. These findings highlight a critical gap between adolescents’ training experiences and the perceptual and decision-making demands of cycling environments. The current findings therefore underscore the urgent need to develop evidence-based traffic safety programmes that explicitly incorporate HP training for adolescent bicyclists, with content differentiated by hazard type and tailored to the cognitive developmental stage of the target age group. Policymakers and practitioners should consider integrating such programmes into the existing middle school curriculum in China, ensuring that both EP and BP hazards receive dedicated instructional attention. Complementing classroom-based instruction with digital tools, such as mobile applications or interactive simulation platforms, could provide adolescent bicyclists with repeated, hands-on exposure to a diverse range of real-world cycling hazards in a controlled and scalable format.

## 5. Conclusions

Drawing on a video-based hazard prediction task administered to adolescent bicyclists across three middle school grade levels, this study demonstrates that students in the ninth and eighth grade outperformed seventh graders in hazard perception and making safe decisions on BP hazards, although students in the ninth grade made more safe decisions on the EP hazards than the seventh and eighth graders. Importantly, this study also reveals that the relationships between hazard perception ability and decision-making accuracy of adolescent bicyclists vary with hazard types. Accordingly, these results underscore the necessity for targeted hazard-specific HP training programmes for middle school–aged bicyclists in China, ensuring that road safety education is responsive to the distinct perceptual and decision-making demands imposed by different hazard types across different stages of adolescent cognitive development.

## Figures and Tables

**Table 1:** Demographic information of the participants (*n* = 115).

Variables		Total (*n* = 115)	7th Grade (*n* = 40)	8th Grade (*n* = 38)	9th Grade (*n* = 37)	Statistic
Gender, *n* (%)	Male	61 (53.0)	24 (60.0)	19 (50.0)	18 (48.6)	*χ*^2^ = 1.21
	Female	54 (47.0)	16 (40.0)	19 (50.0)	19 (51.4)	
Age (years)	M ± SD	14.22 ± 1.05	13.25 ± 0.67	14.18 ± 0.39	15.30 ± 0.78	*F*(2, 112) = 100.47 **
	Min–Max	13–16	13–16	14–15	13–16	
Bicycling frequency, *n* (%)	1–2 days/week	13 (11.3)	11 (27.5)	2 (5.3)	0 (0)	*F*(2, 112) = 11.39 **
	3–4 days/week	67 (58.3)	24 (60.0)	22 (57.9)	21 (56.8)	
	5–6 days/week	35 (30.4)	5 (12.5)	14 (36.8)	16 (43.2)	
Bicycling distance (km)	M ± SD	176.38 ± 283.13	93.40 ± 219.65	228.82 ± 348.93	212.24 ± 254.12	*F*(2, 112) = 2.75
	Min–Max	7–1395	8–1395	7–1200	10–1200	
Crash history	M ± SD	0.25 ± 0.62	0.18 ± 0.64	0.26 ± 0.55	0.32 ± 0.67	*F*(2, 112) = 0.56
	Min–Max	0–3	0–3	0–2	0–2	

Note. ** *p* < 0.01.

**Table 2:** Correlations between the four questions and bicycling related data (*n* = 115).

Variables	1	2	3	4	5	6	7	8	9
1. Bicycling distance	1	0.60 **	0.25 **	0.10	0.22 **	0.18	0.12	−0.11	0.17
2. Bicycling frequency		1	0.23 *	0.16	0.25 **	0.24 *	−0.06	−0.12	0.01
3. HP (BP)			1	0.64 **	0.36 **	0.36 **	0.21 *	0.05	0.24 **
4. DM (BP)				1	0.30 **	0.25 **	0.10	0.24 **	0.09
5. HP (EP)					1	0.53 **	−0.09	−0.13	−0.08
6. DM (EP)						1	0.01	−0.03	0.10
7. Bicycling ability							1	0.45 **	0.68 **
8. Awareness of others								1	0.23 *
9. Self-confidence in bicycling									1

Note. * *p* < 0.05, ** *p* < 0.01.

## Data Availability

The original data presented in the study are openly available on [FigShare] at [10.6084/m9.figshare.31648156].

## Supplementary Materials

The following supporting information can be downloaded at https://www.mdpi.com/article/10.3390/bs16050748/s1.
