# Supplementary material for: Effects of Hazard Types on Hazard Perception and Decision-Making Among Adolescent Bicyclists: Results of a Hazard Prediction Task
Source: Behav Sci (Basel). 2026 May 12;16(5):748. doi: 10.3390/bs16050748 (PMC13203711; doi:10.3390/bs16050748)
Supplement: Supplementary file 1 [file behavsci-16-00748-s001.zip › behavsci-4223866-supplementary.pdf]

Clip	Length (s)	Hazard type	Brief description	Screenshot
1	8	BP	A bus ahead stopped in this lane, blocking the road ahead	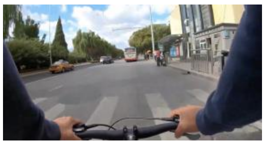
2	6	BP	A black car in front with its taillights on and braking	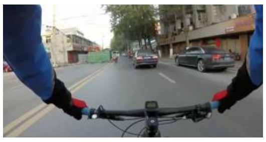
3	5	EP	A blue electric bicycle appeared	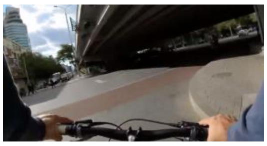
4	9	EP	A white car emerged from the intersection on the right	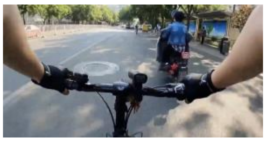
5	5	EP	A bus blocked the cyclist’s view on the left, while the pedestrian in front wanted to cross the road	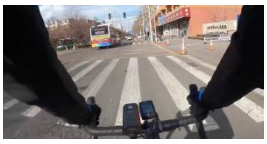
6	6	BP	The white car in front started to drive in front of the camera bicycle	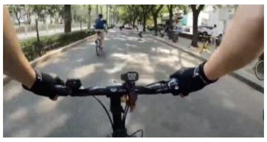
7	7	EP	The first white car blocked the cyclist’s view on the left, while the pedestrian in front wanted to cross the road	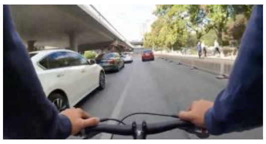
8	7	EP	A yellow taxi suddenly appears at the intersection directly ahead	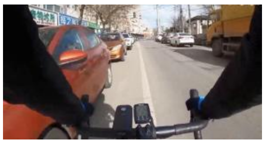

9	8	BP	The yellow engineering vehicle in front was navigating road construction	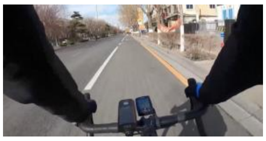
10	6	EP	A pedestrian in the front and to the left quickly crossed the road	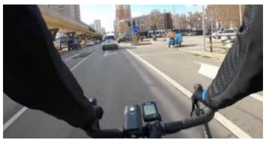
11	5	BP	Some pedestrians disembarked the bus directly in front	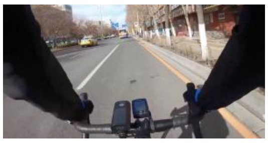
12	9	BP	The driver of the black car directly ahead opened the door to get out of the car	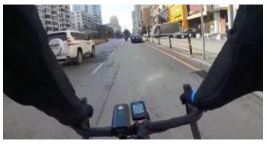
13	5	BP	A white car in front and to the left turned right into the trail	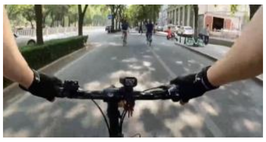
14	6	BP	A silver car in the left lane merged right into this lane	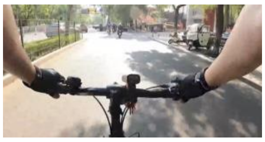
15	7	EP	A bicycle entered the merge lane from the front and to the right	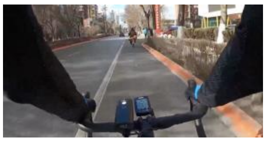
16	6	EP	A white car in front with its taillights on and braking	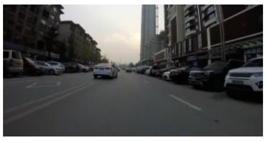
